# Myosteatosis can Predict Unfavorable Outcomes in Advanced Hepatocellular Carcinoma Patients Treated With Hepatic Artery Infusion Chemotherapy and Anti-PD-1 Immunotherapy

**DOI:** 10.3389/fonc.2022.892192

**Published:** 2022-05-16

**Authors:** Xiaoping Yi, Yan Fu, Qianyan Long, Yazhuo Zhao, Sai Li, Chunhui Zhou, Huashan Lin, Xiaolian Liu, Chang Liu, Changyong Chen, Liangrong Shi

**Affiliations:** ^1^ Department of Radiology, Xiangya Hospital, Central South University, Changsha, China; ^2^ National Clinical Research Center for Geriatric Disorders (Xiangya Hospital), Central South University, Changsha, China; ^3^ Hunan Key Laboratory of Skin Cancer and Psoriasis, Xiangya Hospital, Changsha, China; ^4^ Hunan Engineering Research Center of Skin Health and Disease, Xiangya Hospital, Changsha, China; ^5^ Department of Dermatology, Xiangya Hospital, Central South University, Changsha, China; ^6^ Department of Pharmaceuticals Diagnosis, GE Healthcare, Changsha, China

**Keywords:** myosteatosis, predictor, treatment response, hepatocellular carcinoma, hepatic artery infusion chemotherapy, anti-PD-1 immunotherapy

## Abstract

**Aim:**

To evaluate the feasibility of computed tomography (CT) - derived measurements of body composition parameters to predict the risk factor of non-objective response (non-OR) in patients with hepatocellular carcinoma (HCC) undergoing anti-PD-1 immunotherapy and hepatic artery infusion chemotherapy (immune-HAIC).

**Methods:**

Patients with histologically confirmed HCC and treated with the immune-HAIC were retrospectively recruited between June 30, 2019, and July 31, 2021. CT-based estimations of body composition parameters were acquired from the baseline unenhanced abdominal CT images at the level of the third lumbar vertebra (L3) and were applied to develop models predicting the probability of OR. A myosteatosis nomogram was built using the multivariate logistic regression incorporating both myosteatosis measurements and clinical variables. Receiver operating characteristic (ROC) curves assessed the performance of prediction models, including the area under the curve (AUC). The nomogram’s performance was assessed by the calibration, discrimination, and decision curve analyses. Associations among predictors and gene mutations were also examined by correlation matrix analysis.

**Results:**

Fifty-two patients were recruited to this study cohort, with 30 patients having a OR status after immune-HAIC treatment. Estimations of myosteatosis parameters, like SM-RA (skeletal muscle radiation attenuation), were significantly associated with the probability of predicting OR (*P*=0.007). The SM-RA combined nomogram model, including serum red blood cell, hemoglobin, creatinine, and the mean CT value of visceral fat (VFmean) improved the prediction probability for OR disease with an AUC of 0.713 (95% CI, 0.75 to 0.95) than the clinical model nomogram with AUC of 0.62 using a 5-fold cross-validation methodology. Favorable clinical potentials were observed in the decision curve analysis.

**Conclusions:**

The CT-based estimations of myosteatosis could be used as an indicator to predict a higher risk of transition to the Non-OR disease state in HCC patients treated with immune-HAIC therapy. This study demonstrated the therapeutic relevance of skeletal muscle composition assessments in the overall prediction of treatment response and prognosis in HCC patients.

## Introduction

Hepatocellular carcinoma (HCC) is considered the third leading cause of cancer-related mortalities worldwide (1). In the case of unresectable HCCs, transcatheter arterial chemoembolization (TACE) is considered the first-line treatment to combat the tumor outgrowth by restricting the blood and nutrient supply to the tumor. Despite the minimally invasive nature of TACE and satisfactory success rate, patients may show the disease progression after TACE, and most importantly, patients who cannot tolerate the TACE procedure due to the portal vein thrombosis often exhibit extremely poor prognostic outcomes. For these patients, administration of FOLFOX (fluorouracil, leucovorin, and oxaliplatin) through hepatic artery infusion chemotherapy (HAIC) has been commonly practiced owing to its higher response rates and improved survival outcomes compared with the sorafenib-based standard systemic treatment (2). However, outcomes of patients treated with HAIC alone or in combination with sorafenib were unsatisfactory (median overall survival: 10.8 - 14.5 months) (3–5).

Recently, HAIC combined with checkpoint blockade immunotherapy (CBI) is proposed to benefit patients with advanced HCC, since HAIC can reduce the tumor burden and induce immunogenic cell death to activate the host anti-tumor immunogenicity (6, 7). The modality of immune-HAIC has shown promising anti-tumor activities in advanced-stage HCC patients (8–10). However, about 40-60% of HCC patients ultimately progress to the non-objective response (Non-OR) disease after immune modulation-HAIC treatment. With the lack of effective treatment and growing demand for alternative aggressive cancer therapy for patients with Non-OR disease, early identification and diagnosis would be essential for formulating an efficient HCC management with a satisfactory prognosis.

To date, a huge gap between the knowledge of predictive biomarkers and the treatment response in HCC patients receiving immuno-HAIC therapy remains the major limitation. Several risk factors associated with poor response rates in HCC patients undergoing HAIC have been identified, including liver cirrhosis and hepatitis B virus (HBV) infection (11). However, due to occurrences of overlapping clinical factors among HCC patients with varying responses at baseline, effective prediction of individualized immune-HCC success rate in HCC patients has not been possible. Preliminary screening of HCC patients at high risk of progressing to the Non-OR status is an unmet need to predict the chance of aggressive treatment failure in this subset of patients.

Myosteatosis refers to abnormal distributions of adipose tissues between and within muscle cells, leading to excessive fat deposition in the muscle, a pathological situation associated with decreased muscle quality, limb function, and physical fitness. Myosteatosis is evaluated on the conventional computed tomography (CT) images, using the radiological characterization of skeletal muscle radiation attenuation (SM-RA) (12–14). A growing body of evidence suggests myosteatosis as the negative indicator of poor treatment response and prognosis in several cancers, including HCC (15). However, there is limited understanding of the clinical impact of assessing the baseline body composition, for example, CT-derived myosteatosis, in HCC patients undergoing immune-HAIC therapy.

Therefore, this study evaluated the clinico-pathological implication of myosteatosis to abdominal CT images collected to determine treatment responses in HCC patients. We hypothesized that the CT-based evaluation of myosteatosis might be effective in predicting the probability of objective response (OR) to immune-HAIC in HCC patients prior to the treatment initiation. Early screening of HCC patients to determine the risk of Non-OR development and the urgent necessity of alternative aggressive therapy could be beneficial in reducing the HCC mortality rate.

## Patients and Methods

### Study Design and Patients

Patients with histologically confirmed HCC between June 30, 2019, and July 30, 2021, were retrospectively identified from our hospital. Patient inclusion criteria were as follows: 1) age ≥18 years; 2) had portal vein invasion; 3) had disease progression or were intolerant to one or more systemic treatments with antiangiogenic tyrosine kinase inhibitors (TKIs); 4) an Eastern Cooperative Oncology Group (ECOG) performance score of 0-1; 5) belonged to Child Pugh Class A or B (score =7).While patients having coexisting non-HCC malignancies, missing or suboptimal CT images, or missing complete clinical data were excluded from this study.

**Figure 1:**
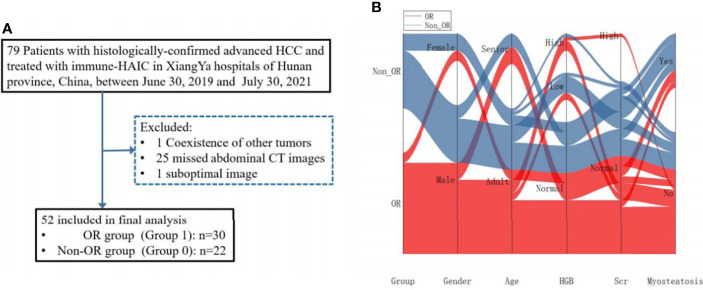
**(A)** The flow-chart illustrating the recruiting procedure for hepatocellular carcinoma (HCC) patients. **(B)** Sankey diagram exhibiting projections of outcomes between the groups, gender, age, HGB, Scr, as well as Myosteatosis.

SM-RA measurements for myosteatosis were acquired from the baseline pre-enhanced abnormal CT images at the L3 vertebra level to build the predictive model of the risk of treatment failure. Descriptions of patient selection/recruitment and applied exclusion criteria are shown in **Figure 1**. This single-centered retrospective study protocol was approved by the Medical Ethics Committee of the Xiangya Hospital.

### Data Collection

Information on the abdominal CT images, demographics, laboratory test results, clinical records, outcome data were retrieved from our hospital medical record archives. Only the available baseline data were included for analysis. All data were thoroughly reviewed by two independent, experienced clinicians (SL and YZ) who were blind to the clinical and pathological information of study subjects. A third reviewer (LS) was introduced to adjudicate any differences in interpretations between the two primary reviewers.

### Treatment, Tumor Response Assessments, and Follow-Up

HAIC was conducted as reported previously (2). The femoral artery puncture following TACE was performed in every treatment cycle. FOLFOX was administered *via* a 2.7 French microcatheter connected to the feeding arteries of the tumor and associated thrombus at the following doses: on day 1, oxaliplatin at 85 mg/m^2^ from 0 - 2 h; leucovorin at 400 mg/m^2^ from 2 - 3 h, and 5-fluorouracil at 400 mg/m^2^ bolus at 3 h, then at 2400 mg/m^2^ over 46 h. PD-1 inhibitors, including pembrolizumab, camrelizumab, and toripalimab, were administrated intravenously within 3 days after HAIC. Decisions on the dose adjustment of FOLFOX, disruption or discontinuation of HAIC and/or PD-1 therapy were made at the discretion of the investigator based on the patient’s clinical status.

Patients’ radiological responses were assessed per hepatocellular carcinoma-specific modified RECIST guidelines (16) such as at the interval of every 6 to 8 weeks. Two experienced radiologists (XY, and LS with 11 and 20 years’ experience, respectively), determined the tumor responses by consensus. In case of any discrepancy in the opinion between the two primary radiologists, a third radiologist (CC, with more than 30 years of experience in abdominal radiology) was introduced to determine the final tumor response by consensus.

The objective response (ORR) and disease control (DCR) rates were recorded. Progression-free survival (PFS) was defined as the time period between the treatment initiation and disease advancement or death. Overall survival (OS) was defined by the time period between the treatment initiation and cancer-related death.

### Analysis of Abdominal CT Images and the Measurement of Body Composition

Patients’ baseline abdominal CT scans taken prior to the initial treatment were retrieved from the PACS (Picture Archiving and Communication Systems, Carestream, Canada) of our hospital. The time length between the baseline abdominal CT imaging and treatment was 1 to 14 days. Reconstruction of all axial CT images was made to a uniform thickness of 1 mm.

One axially unenhanced image from the set of each abdominal CT scan at the L3 level was included. Back and abdominal wall muscles including the paraspinal, transversus abdominis, psoas, rectus abdominis, external and internal oblique muscles were segmented (manually) for each scan on the specific axial image. Then body compositions were measured on each of the segmented images using the ImageJ software (National Institutes of Health).

**Figure 2:**
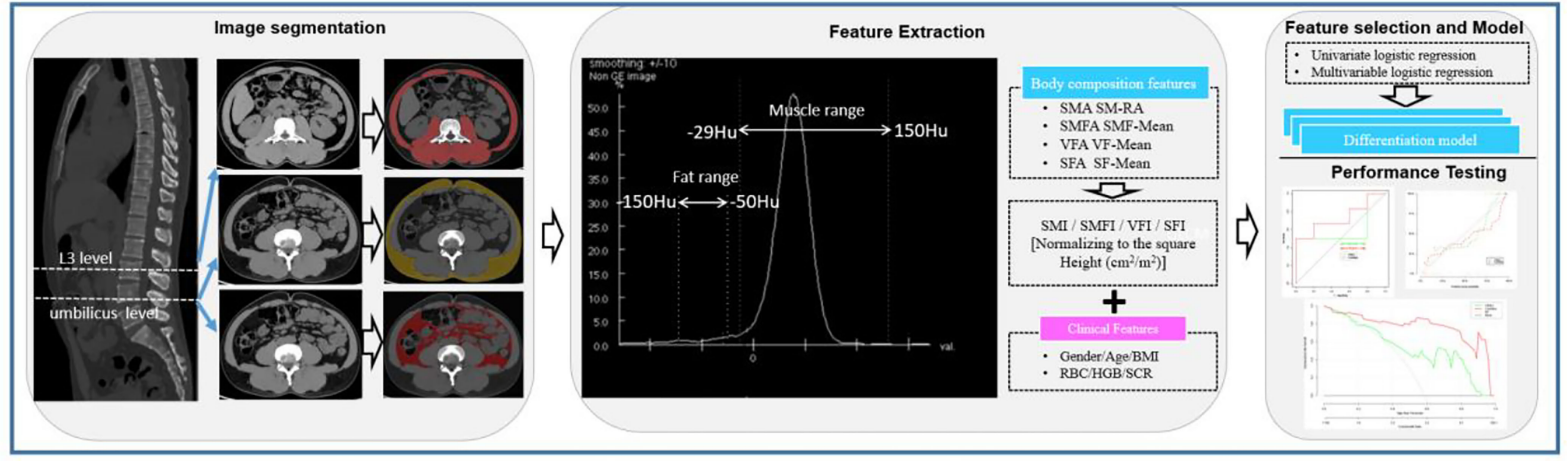
Workflow for unenhanced abdominal CT images segmentation, feature extraction, selection, modeling, and performance testing for this study. (I) Segmentation of CT images. The third lumbar vertebra (L3) and umbilical level were selected for the body composition feature extractions. (II) Strategy for extracting body composition features from selected CT images. (III) Feature selection and prediction model construction with the univariate and multivariate logistic regressions based on the body composition and clinical features. Receiver operating characteristic (ROC) and calibration curves were applied to assess the model performance. Decision curve analysis was carried out to evaluate the clinical values of prediction models. L3 level, the third lumbar vertebra; SMA, skeletal muscle area; SM-RA, skeletal muscle radiation attenuation; SMFA, skeletal muscle fat area; SMF-Mean, skeletal muscle fat mean; VFA, visceral fat area; VF-Mean, visceral fat mean; SFA, subcutaneous fat area; SF-Mean, subcutaneous fat mean; SMI, skeletal muscle index; SMFI, skeletal muscle fat index; VFI, visceral fat index; SFI, subcutaneous fat index; RBC, red blood cell; HGB, hemoglobin; Scr, serum creatinine.

Multiple body composition measurement parameters such as the skeletal muscle area (SMA) (-29 to -150Hu), visceral fat area (VFA) (-150 to -50Hu), subcutaneous fat area (SFA) (-150 to -50Hu), skeletal muscle fat area (SMFA) (-150 to -30Hu), SM-RA, as well as the mean CT values of SMA (SM-Mean), VFA (VF-Mean), SFA (SF-Mean), SMFA (SMF-Mean) were calculated following the previously reported method (17). Subsequently, the skeletal muscle fat index (SMFI) and skeletal muscle index (SMI) were estimated with the normalization of the measured muscle area to the square height (cm^2^/m^2^). The CT imaging analysis and corresponding prediction modeling are illustrated in **Figure 2**.

Sarcopenia referred to the sex-specific cut-off points (Men: 52.4 cm^2^/m^2^; Women: 38.5 cm^2^/m^2^) for L3 SMI (18). Because of the unavailability of any established cutoff value for myosteatosis diagnosis, we determined the cut-off value of myosteatosis for the L3 SMI based on the gender stratified tertiles, which were adjusted to the lowest SM-RA tertile for the myosteatosis diagnosis.

### Testing the Reproducibility of CT Body Composition Estimations

Thirty randomly picked abdominal CT images were independently segmented by two expert radiologists. The intra-observer (reader 1 two times) and inter-observer (reader 1 vs. reader 2) correlation coefficients (ICC) were calculated. The final consistency was assessed following the criteria applied to the ICC value, such as poor reproducibility at <0.20, fair reproducibility at 0.21–0.40, moderate reproducibility at 0.40–0.60, good reproducibility at 0.61–0.80, and excellent reproducibility at 0.81–1.00 values. The CT body composition measurement ICC (Inter-) values were ranged between 0.977 and 1.000, while that for the intra-observer ICC was ranged from 0.983 to 1.000. Hence, reader 1’s body composition measurements were included in the subsequent analyses.

### Risk Factors Associated With Treatment Response to Immunotherapy

Univariate and multivariable logistic regressions were executed to explore associations between risk factors and treatment responses. For survival analysis, Cox proportional hazard modeling was applied to reveal the connection between myosteatosis and PFS/OS in HCC patients. Adjusted odd ratio (OR) and hazard ratio (aHR) with 95% confidence interval (CI) were calculated to assess the effect size. Survival differences between patients with or without myosteatosis were compared using the log-rank test. Additionally, the correlation analyses between SM-RA and other clinical parameters were also performed.

### An Individualized Prediction Model Development

The univariate logistic regression was conducted to evaluate the association between clinical/laboratory variables and myosteatosis, and risks of unfavorable outcomes. The univariate analysis (*P*<0.05) identified the predictor candidates, then the multivariate logistic regression analysis, including the likelihood ratio test, with Akaike’s information criterion (AIC) as the stopping rule was applied to select the correlated factors. The optimal combination of factors was well-correlated with AIC minimums.

The numbers of predictors were set to <1/10-1/3 of the dependent group’s number to control the overfitting. The number of potential features was restricted to not more than 6 for treatment-response predictions (20 Non-ORs). A model for predicting the probability of OR based on clinical data excluding myosteatosis parameters was developed. Subsequently, another model including myosteatosis parameters combined with the clinical model was constructed. A nomogram was created to support clinicians with an efficient quantitative tool in predicting the individual probability of response risk. Comparisons of performance data for these two models were carried out.

### The Nomogram Validation and Model Performance Test

The nomogram was calibrated by evaluating calibration curves (Hosmer-Lemeshow H test), and the receiver operating characteristic (ROC) curve was plotted to assess the diagnostic efficiency. The final predictive performances of the two models were examined using the 4-fold cross-validation strategy, and the average performance of the model was presented as the cross-validated performance. A 1000-iteration bootstrap analysis was performed for the proposed model to estimate the prediction error. A random subset of 70% of patients from either the validation or training cohort was selected for each repetition, and the respective AUC values were determined. The clinical applications of the nomogram were evaluated using the decision curve analysis in the validation cohort.

### Next-Generation Sequencing

The mutation status of targeted genes was determined by next-generation sequencing using tumor sample (Foundation Medicine, Cambridge, Massachusetts, USA). The targeted DNA library comprising 425 genes for panel sequencing was constructed by blood-based circulating tumor DNA next-generation sequencing (Nanjing Shihe Jiyin Biotechnology Inc. Nanjing, China). In brief, extracted tumor genomic DNA was fragmented into 300~350bp using Covaris M220 instrument (Covaris). Sequencing libraries were prepared with KAPA Hyper Prep kit (KAPA Biosystems) with optimized protocols.

### Analyses of Interrelationships Between Body Composition, Clinical and Genetic Features

The association between the identified significant body composition features and clinical features or genetic features was also examined using the correlation matrix analysis (Pearson or Spearman analysis). The scanter plot and heat-map plot were drawn, respectively.

### Statistical Analysis

Statistical analyses were conducted using the R software (v3.5.2; http://www.Rproject.org) or SPSS v22.0 (IBM, United States). Univariate analysis for clinical features was executed by the chi-squared (χ^2^) test for categorical variables or the Mann-Whitney U test for continuous variables, as appropriate to compare differences between the patients with non-severe and severe illnesses. The calibration plotting and nomogram construction were conducted using the “rms” package (R software). Two-sided statistical significance analyses were performed with the cut-off *P*=0.05.

## Results

### Patient Characteristics

Fifty-two patients receiving HAIC combining anti-PD-1 immunotherapy were included in this study. Amongst them, 30 patients had the OR disease, including one complete response (CR) and 29 partial responses (PR). The ORR was 57.7%. The remaining 22 patients exhibited the Non-OR status, including 10 with stable disease (SD) and 12 with progressive disease (PD).

Significant differences between the OR and Non-OR groups were detected for several laboratory biomarkers, including hemoglobin (HGB), red blood cells (RBC), serum, and creatinine (Scr) level (**Table 1**). There were no statistically significant differences between the effective and invalid groups in terms of other clinical variables.

**Table 1:** Demographic, clinical, laboratory, pathologic and body composition characteristics of 52 HCC patients.

Characteristic	Total (n=52)	Objective remission (n=30)	Non-objective remission(n=22)	*P* Value	Myosteatosis(n=16)	Non-myosteatosis(n=36)	*P* Value
Demographics and clinical characteristics							
Gender (n, %)				0.209			0.701
Male	44 (84.6)	27 (90)	17 (77.3)		14 (87.5)	30 (83.3)	
Female	8 (15.4)	3 (10)	5 (22.7)		2 (12.5)	6 (16.7)	
Age (y)^#^	50 (42-58)	50 (45-57)	49 (39-58)	0.824	58 (51-68)	47 (39-54)	0.001
BMI^#^	24.1 (22.5-26.6)	23.6 (22.5-26.9)	24.3 (21.8-26.7)	0.817	24.9 (23.1-28.1)	23.5 (22.4-26.3)	0.378
HBV Infection (n, %)				0.584			0.412
Yes	41 (80.8)	25 (83.3)	17 (77.3)		14 (87.5)	28 (77.8)	
No	10 (19.2)	5 (16.7)	5 (22.7)		2 (12.5)	8 (22.2)	
Child-Pugh (n, %)				0725			0.412
A	42 (80.8)	25 (83.3)	17 (77.3)		14 (87.5)	28 (77.8)	
B	10 (19.2)	5 (16.6)	5 (22.7)		2 (12.5)	8 (22.2)	
Port vein invasion (n, %)				0.971			0.842
Vp1-2	5 (9.6)	3 (10.0)	2 (9.1)		2 (12.5)	3 (8.3)	
Vp3	25 (48.1)	14 (46.7)	11 (50.0)		8 (50.0)	17 (47.2)	
Vp4	22 (42.3)	13 (43.3)	9 (40.9)		6 (37.5)	16 (44.4)	
Extrahepatic site (n, %)				0.526			1.000
Absent	40 (76.9)	22 (76.7)	18 (77.3)		12 (75.0)	28 (77.8)	
Present	12 (23.1)	8 (23.3)	4 (22.7)		4 (25.0)	8 (22.2)	
Laboratory findings							
Blood routine test							
WBC (10^9^/L)^#^	4.7 (3.2 -5.9)	4.5 (3.5-6.3)	4.7 (2.9-5.6)	0.863	4.6 (3.1-5.5)	4.7 (3.3-6.3)	0.736
RBC (10^9^/L)^#^	4.2 (4.0 -4.7)	4.3 (4.1-4.8)	4.1 (3.9-4.37)	0.044	4.1 (3.9-4.4)	4.2 (4.16-4.8)	0.177
HGB (g/L)^#^	130.0 (118.8-144.3)	131.5 (127.0-148.8)	128.0 (112.3-133.0)	0.014	128.5 (117.0-134.8)	131.0 (121.5-150.3)	0.201
PLT (10^9^/L)^#^	122.5 (78.3-206.0)	124.0 (64.5-198.0)	119.0 (88.5-225.8)	0.560	119.0 (76.3-184.3)	124.0 (78.3-232.5)	0.781
Liver function test							
TP (g/L)^#^	68.0 (63.4-72.9)	68.0 (63.6-71.8)	67.0 (63.1-74.9)	0.889	65.2 (65.8-71.0)	69.5 (65.3-74.9)	0.113
Albumin (g/L)^#^	37.6 (34.1-40.8)	37.6 (34.6-41.8)	37.4 (31.3-39.3)	0.274	35.6 (31.7-38.9)	38.0 (34.5-41.9)	0.129
Globulin (g/L)^#^	30.1 (27.0-34.3)	29.2 (27.4-32.7)	33.1 (25.9-41.4)	0.295	29.2 (25.5-34.1)	30.8 5(27.0-34.7)	0.804
ALT (U/L)^#^	42.3 (28.4-62.2)	45.6 (29.6-72.7)	33.7 (25.8-52.6)	0.082	46.6 (29.9-61.6)	39.0 (27.3-62.2)	0.378
AST (U/L)^#^	65.8 (37.9-89.8)	61.6 (38.2-84.6)	74.3 (36.4-100.1)	0.493	78.2 (52.6-146.2)	61.2 (5.1-79.4)	0.052
Tbile (μmol/L)^#^	15.7 (10.5-23.7)	15.9 (10.0-24.1)	15.0 (11.3-23.8)	0.817	19.6 (13.3-27.9)	14.2 (10.0-21.9)	0.132
Scr (mg/dl)^#^	75.5 (65.0-90.6)	82.5 (66.0-93.3)	70.9 (57.9-81.9)	0.022	68.5 (58.2-82.6)	77.4 (66.0-91.0)	0.102
AFP							
<400 ng/mL	21 (40.4)	13 (43.3)	8 (36.4)		6 (37.5)	15 (41.7)	
≥400 ng/mL	31 (59.6)	17 (56.7)	14 (63.6)		10 (62.5)	21 (58.3)	
CT-based body composition							
SMFmean^#^	-56.8 (-59.5-54.3)	-56.3 (-58.8-54.6)	-58.2 (-61.5-53.4)	0.259	-56.8 (-59.1-54.3)	-56.9 (-59.9-54.7)	0.968
Myomean^#^	12.3 (9.8-13.9)	12.5 (9.8-13.4)	12.2 (9.7-14.5)	0.970	11.0 (9.8-12.7)	12.8 (9.8-14.5)	0.104
VFmean^#^	-92.3 (-96.4-85.2)	-95.1 (-97.8-87.9)	-89.6 (-95.2-82.6)	0.036	-89.8 (-95.8-83.6)	-97.3 (-101.4-91.7)	0.648
SFmean^#^	-97.4 (-101.6-91.3)	-98.2 (-102.1-93.4)	-94.8 (-101.4-88.6)	0.236	-97.7 (-104.0-89.8)	-97.3 (-101.4-91.7)	0.706
SMFI^#^	1.4 (0.7-2.5)	1.1 (0.6-1.9)	1.5 (0.8-3.2)	0.115	2.5 (1.5-3.1)	0.9 (0.6-1.6)	0.0005
SMI^#^	42.4 (39.1-47.5)	45.1 (40.5-48.2)	40.8 (31.6-45.2)	0.078	41.1 (33.1-52.5)	42.7 (39.2-47.2)	0.565
VFI^#^	40.1 (28.7-53.1)	41.6 (27.9-57.2)	39.7 (31.4-50.4)	0.767	41.0 (33.1-52.5)	39.3 (27.3-53.2)	0.341
SFI^#^	51.3 (35.4-69.2)	50.6 (30.6-63.2)	51.3 (35.8-80.2)	0.517	51.33 (39.0-69.2)	49.3 (31.7-69.0)	0.89
SVR^#^	1.2 (1.0-1.6)	1.1 (0.9-1.5)	1.3 (1.0-2.0)	0.159	1.1 (1.0-1.4)	1.3 (0.9-2.0)	0.394
SM-RA^#^	44.3 (40.2-48.6)	46.1 (43.1-50.1)	41.5 (37.2-45.9)	0.007	37.6 (36.2-40.2)	46.7 (44.0-50.6)	0.000
Sarcopenia, n (%)				0.393			
Yes	45 (86.5)	27 (90)	18 (81.8)		14 (87.5)	31 (86.1)	
No	7 (13.5)	3 (10)	4 (18.2)		2 (12.5)	5 (13.9)	
Myosteatosis, n (%)				0.010			
Yes	16 (30.8)	5 (16.7)	11(50.0)				
No	36 (69.2)	25 (83.3)	11 (50.0)				

Unless otherwise indicated, data are numbers of patients, and data in parentheses are percentages. ^#^represents data was presented as media (IQR, inter-quartile range). BMI, Body Mass Index; SBP, Systolic Blood Pressure; DBP, Diastolic Blood Pressure; HBV, Hepatitis B Virus; WBC, white blood cell; RBC, Red Blood Cell; HGB, Hemoglobin; PLT, Platelet count; TP, Total, Protein; ALT, Alanine aminotransferase; AST, Aspartate aminotransferase; Tbile, Total Bilirubin; Scr, Serum creatinine; AFP, A-fetoprotein; SMFmean, skeletal muscle fat mean; Myomean, Myosteatosis mean; VFmean, visceral fat mean; SFmean, subcutaneous fat mean; SMFI, skeletal muscle fat index; SMI, skeletal muscle index; VFI, visceral fat index; SFI, subcutaneous fat index; SVR, Surface-Volume Ratio; SM-RA, skeletal muscle radiation attenuation.

### Myosteatosis Measurements in Patients With OR and Non-OR Disease

**Figure 3:**
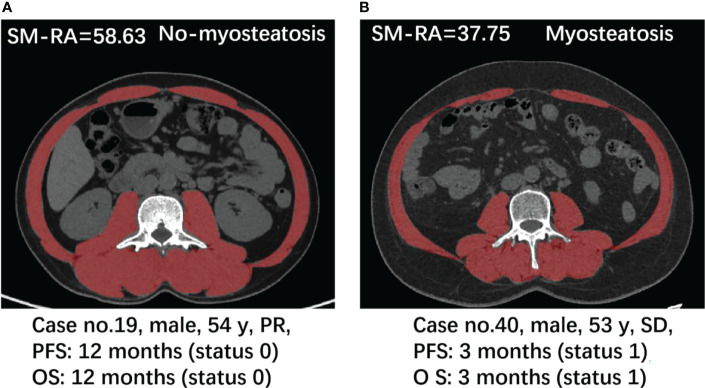
Case presentation. Axial CT images at the L3 level used for the measurement of multiple parameters for body composition, respectively in a 54-year-old man with PR **(A)** and a 53-year-old man with SD **(B)**.

Body composition features of all patients are shown in **Table 1**. In comparison to the Non-OR group, the OR patients exhibited a significantly higher skeletal muscle density (median SM-RA: 46.05 Hu vs. 41.47 Hu), and lower incidences of myosteatosis (16.7% vs. 50.0%) with *P*=0.007 (**Table 1**). The above differences were also reflected in the following two specific cases (**Figure 3**). Moreover, OR patients demonstrated a lower VF density (mean: -95.07 Hu vs. -89.61 Hu). There were no significant differences between the two groups in the remaining body composition parameters, such as SMI and the incidence of sarcopenia.

Patient characteristics for the comparison between patients with or without myosteatosis status are described in **Table 1**.

### Association of SM-RA With OR, OS and PFS in HCC

To further access the association of SM-RA with the treatment response and prognosis in HCC patients receiving HAIC combined with PD-1 immunotherapy, binary logistic regression and Cox proportional hazard models were constructed. Our results (**Tables 2**–**4**) demonstrated that a lower SM-RA was an independent risk predictor for treatment failure, shorter PFS and OS, in this cohort of HCC patients, after adjusting all other risk factors, including gender, age, HGB, Scr, and VFmean (AOR=1.21, 95% CI: 1.02-1.44, *P*=0.033; AHR for PFS=0.92, 95%CI: 0.85-0.99, *P*=0.023; AHR for OS=0.91, 95%CI: 0.84-0.98, *P*=0.012). We also found that a higher VFmean value at baseline was an independent indicator for OS in this cohort.

**Table 2:** Associations of lower SM-RA with treatment response of HAIC combined with immunotherapy in 52 patients with advanced HCC.

Vaiable	Model Ia		Model IIb		Model IIIc	
	AOR (95%CI)	*P*	AOR (95%CI)	*P*	AOR (95%CI)	*P*
SM-RA	1.258 (1.081, 1.463)	0.003	1.203 (1.019 1.420)	0.029	1.209 (1.016, 1.438)	0.033

SM-RA, Skeletal muscle radiation attenuation; HAIC, hepatic artery infusion chemotherapy; AOR, adjusted odd ratio; CI, confidence interval; VFmean, Mean CT value of visceral fat.

a Adjusted for gender and age.

b Additionally adjusted for hemoglobin and Serum creatinine.

c Additionally adjusted for VFmean.

**Table 3:** Associations of lower SM-RA with progression free survival (PFS) of HAIC combined with immunotherapy in 52 patients with advanced HCC.

Vaiable	Model Ia		Model IIb		Model IIIc	
	AHR (95%CI)	*P*	AHR (95%CI)	*P*	AHR (95%CI)	*P*
SM-RA	0.896 (0.841, 0.954)	0.001	0.902 (0.839, 0.970)	0.005	0.915 (0.848, 0.988)	0.023

SM-RA, Skeletal muscle radiation attenuation; HAIC, hepatic artery infusion chemotherapy; AHR, adjusted hazard ratio; CI, confidence interval.

a Adjusted for gender and age.

b Additionally adjusted for hemoglobin.

c Additionally adjusted for Serum creatinine.

**Table 4:** Associations of lower SM-RA with overall survival (OS) of HAIC combined with immunotherapy in 52 patients with advanced HCC.

Vaiable	Model Ia		Model IIb		Model IIIc	
	AHR (95%CI)	*P*	AHR (95%CI)	*P*	AHR (95%CI)	*P*
SM-RA	0.933 (0.879, 0.990)	0.023	0.896 (0.835, 0.962)	0.002	0.905 (0.837, 0.978)	0.012
VFmean	1.063 (1.007, 1.121)	0.026	1.076 (1.013, 1.142)	0.017	1.072 (1.009, 1.138)	0.017

SM-RA, Skeletal muscle radiation attenuation; HAIC, hepatic artery infusion chemotherapy; AHR, adjusted hazard ratio; CI, confidence interval; VFmean, Mean CT value of visceral fat.

a Before adjusted for other variables.

b Adjusted for gender and age.

c Additionally adjusted for hemoglobin.

### Impact of Myosteatosis on the Performance of Prediction Models

**Figure 4:**
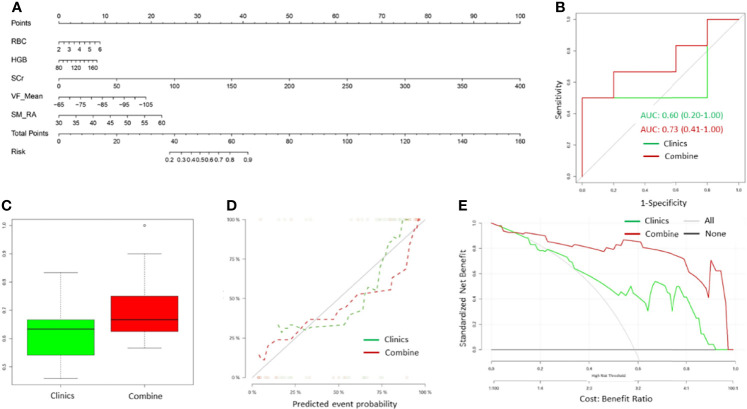
The myosteatosis nomogram, ROC curve, calibration curve, and decision curve for predicting unfavorable outcomes in advanced-stager HCC patients treated with hepatic artery infusion chemotherapy (HAIC) combined with PD-1 immunotherapy. **(A)** Myosteatosis nomogram was constructed in the training cohort, including SM-RA, VFMean, Scr, HGB, and RBC. Risk represents the probability that the patient may obtain ORR after treatment. **(B)** An example of ROC curves for the independent training cohort for both clinical and combined models. **(C)** Box plots showing the AUCs for the clinical and combined models in multiple modeling processes. The green label indicates the clinical model, and the red indicates the combined model. **(D)** Calibration curves of the myosteatosis nomogram for clinical and combined models. **(E)** Decision curve analysis of the myosteatosis nomograms for the combined and clinical model alone. The Y-axis measures the net benefit. The green line represents the decision curve for the clinical model. The red line represents the decision curve for the combined model with the addition of CT myosteatosis features to the clinical model. The black line represents the assumption that no patients had the risk of transitioning to unfavorable outcomes, and the grey line represents all patients who would transition to unfavorable outcomes.

The clinical model developed, including RBC, HGB, Scr, and VFmean achieved a mild efficiency with an average AUC of only 0.62 (95% CI: 0.25-0.94). After the addition of SM-RA to this model, we observed a significant improvement in the performance of the combined prediction model, with an average AUC of 0.711 (95%CI: 0.75-0.95) (**Figures 4A–C**).

The calibration curve of the nomogram revealed significant agreement between predicted and observed values (Hosmer-Lemeshow H test, *P* =0.680, **Figure 4D**). Decision curve analysis demonstrated that if the threshold probability of a doctor or a patient was >5%, application of the nomogram to predict the OR status in HCC patients could add extra benefits than either the diagnose-none or diagnose-all-patients scheme (**Figure 4E**). Importantly, the combined model offered more clinical utilitilizations than the clinical model alone beyond the threshold of~10%.

### Survival Analysis

**Figure 5:**
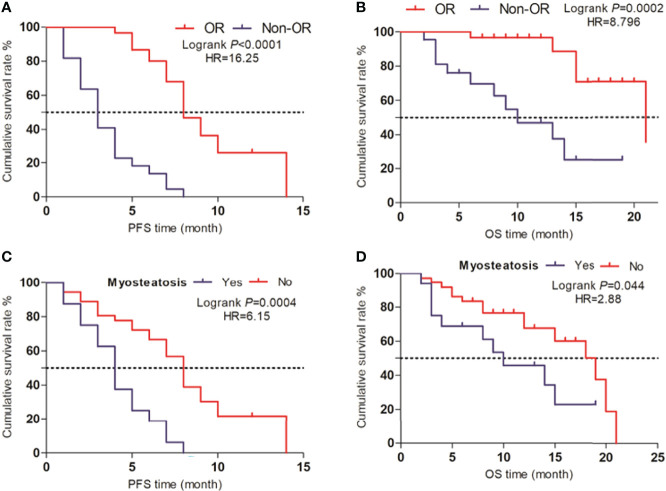
Survival analysis **(A, B)** Life curves showing the PFS and OS time periods between the OR and Non-OR groups. **(C, D)** Life curves showing the PFS and OS time periods between patients with myosteatosis and non-myosteatosis.

The median follow-up period was 10.0 months (IQR: 6.0-13.8, range: 2.0–21.0 months) for all patients. The median PFS and OS were 7.1 and 15.6 months, respectively. Patients with OR demonstrated significantly better PFS (median ± SD, 8.0 ± 0.6 months) and OS (20.0 ± 1.4 months) time when compared to that of Non-OR patents (PFS, 3.0 ± 0.5; OS, 6.0 ± 1.8, months) (**Figures 5A, B**) (*P*<0.001).

For patients without myosteatosis, there were significantly higher PFS (8.0 ± 0.4 months) (*P*<0.001) and OS (19.0 ± 2.8 months) (*P*=0.003) time when compared to patients with myosteatosis (PFS, 4.0 ± 0.5; OS, 9.0 ± 3.4, months) (**Figures 5C, D**).

### Correlation Analysis

**Figure 6:**
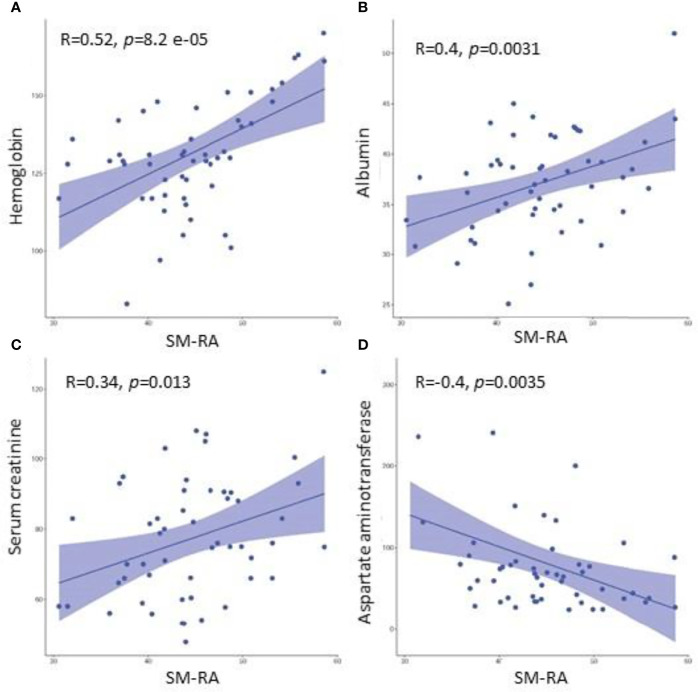
Associations between the SM-RA and clinical parameters (HGB, albumin, Scr, AST). Scatter plots demonstrate that SM-RA is positively associated with HGB **(A)**, albumin **(B)**, and Scr **(C)**, while negatively correlated with AST **(D)**. HGB, hemoglobin; A, albumin; Scr, Serum creatinine; AST, Aspartate aminotransferase.

**Figure 7:**
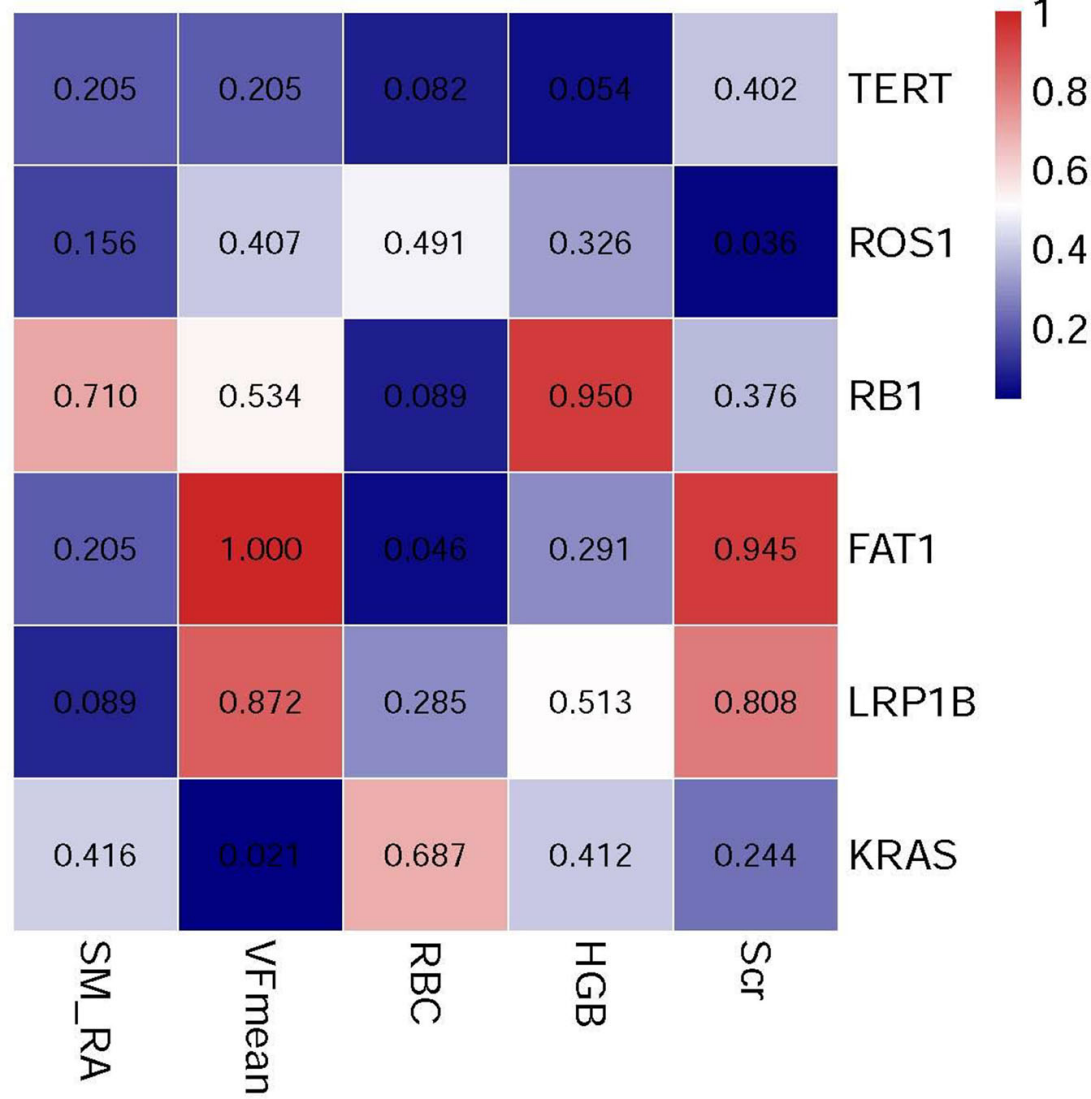
The heat map illustrates associations between the genetic mutation and selected body composition features and clinical variables.

The correlation analysis results indicated that the SM-RA was positively correlated with the serum HGB (r=0.518, *P*<0.001), albumin (r=0.402, *P*=0.003), Scr (r=0.368, *P*=0.007) levels, and negatively correlated with aspartate aminotransferase (AST) (r=-0.398, *P*=0.003) (**Figure 6**). In addition, there is no significant relationship between SM-RA and common tumor-related factors, such as AFP (P=0.121), metastasis (P=0.731) and portal vein invasion (P=0.306). Furthermore, there were significant associations between VFmean and KRAS mutations (r=-0.590, *P*=0.021), RBC count, FAT1 mutation (r=-0.521, *P*=0.046), Scr level, and ROS1 mutation (r=-0.545, *P*=0.036) (**Figure 7**).

**Figure 8:**
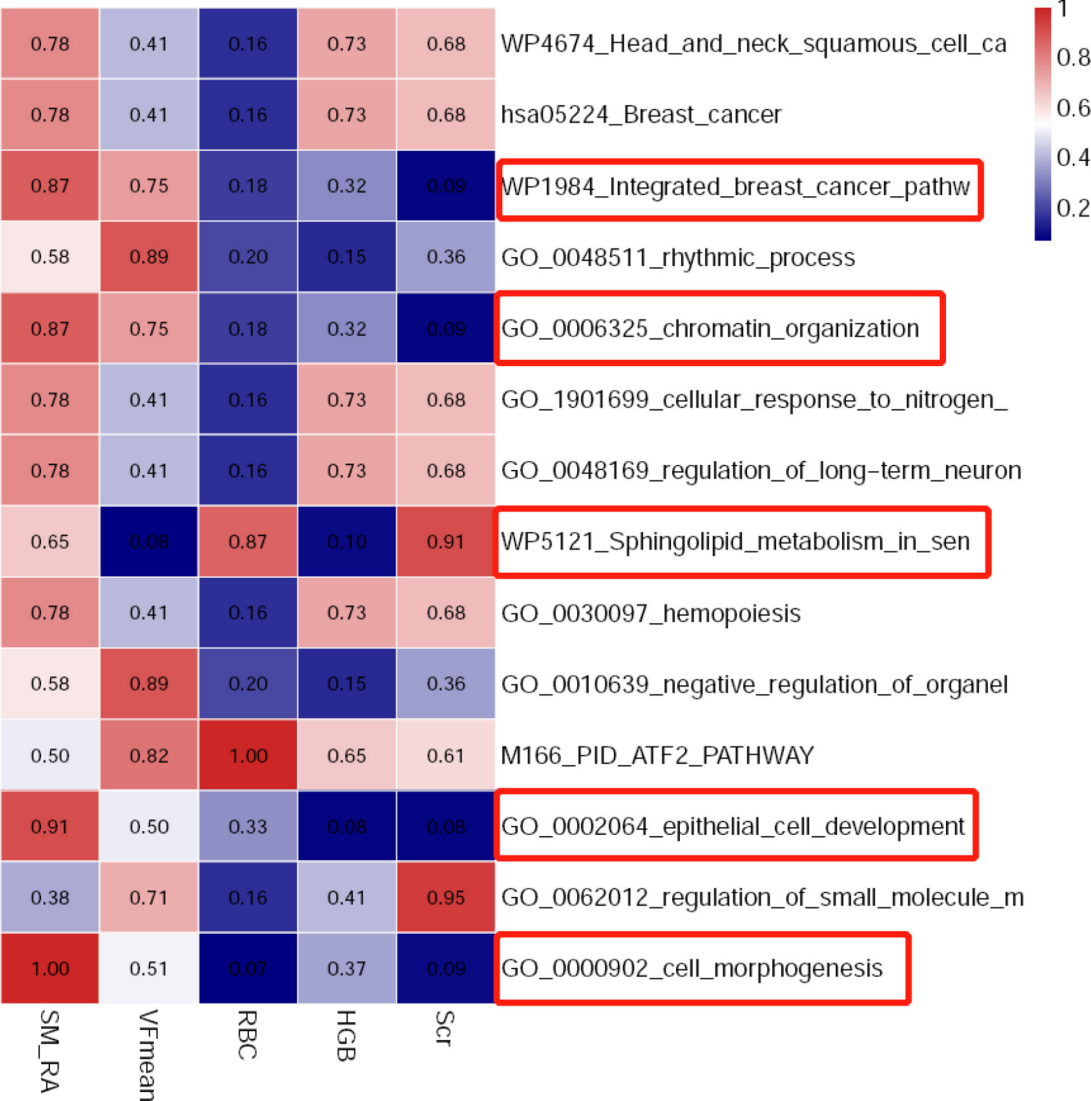
The heat map indicating associations between related biological pathways and selected body composition features and clinical variables.

From the pathway analysis of gene mutations, the following pathways might be associated with these meaningful radiological and clinical features, including cell_morphogenesis, epithelial_cell_development, Sphingolipid_metabolism, chromatin_organization, and Integrated_breast_cancer_pathway (**Figure 8**).

## Discussion

Here, we detected the CT-derived myosteatosis measurement as the independent predictor for OR and prognosis in HCC patients undergoing immune-HAIC therapy. Our combined prediction model and the pre-treatment clinical and laboratory data showed a modest performance level in differentiating patients with a higher probability of OR disease risks from those with lower risks. In summary, this study showed the promising prognostic value of myosteatosis for assessing HCC patients undergoing immune-HAIC treatment in the clinical setting.

Our findings of the importance of abdominal myosteatosis in predicting the OR of HCC patients receiving the immune-HAIC treatment were in line with prior studies reporting adverse effects of myosteatosis in various cancers, including HCC. Myosteatosis is positively associated with accelerated disease severity, higher level of complications, longer hospital stays, worse prognosis, and earlier postoperative recurrence. Recently myosteatosis has been linked to the weak physical state of patients (12), a higher risk of developing severe symptoms (19), and shorter survival (20). Thus, we speculated that patients with myosteatosis of this cohort might have implicated relatively poor physical fitness, resulting in a higher susceptibility to treatment failure.

Moreover, patients in the Non-OR group not only showed significantly higher rates of myosteatosis (54.5% vs. 13.3%) and VF-Mean but also exhibited a higher SMFI and lower SMI values, but not at a statistically significant level, owing to the smaller cohort size. A higher SMFI value indicated enhanced subcutaneous adipose tissue depositions in abdominal muscles, which could lead to obesity, an important underlying etiological factor of myosteatosis. Metabolically active visceral adipose tissue secretes and/or synthesizes many proteins that can lead to the onset of liver carcinoma (21). An increased fat density may represent inflammatory changes in the body. It is reported that the serum inflammatory cytokines’ levels are correlated with survival and OR to various cancer therapies (22). There is a shortage of relevant knowledge about the possible reasons for the association between visceral fat inflammation, immunotherapeutic responses, and poor prognosis of liver cancer, and further explorations need to be continued. Furthermore, the independent association between a lower SMI (sarcopenia) and worse survival in cancer patients has been widely reported, including HCC. We, therefore, hypothesized that patients with myosteatosis might have simultaneously undergone muscular atrophy and obesity as reflected by their higher SMFI values, substantially predisposing them to a higher HCC risk.

The underlying pathophysiological association between the myosteatosis and worse response to immune-HAIC therapy and prognosis in HCC patients has not been explored. In this study, we found a positive correlation between the SM-RA and serological indicators (HGB, albumin, Scr, and AST) in HCC patients, which indicated the nutritional status, organ function, and metabolic status of the body. Recent reports have also shown that skeletal muscle quality (myosteatosis), rather than quantity (sarcopenia), is an independent prognostic marker for a variety of tumorigenesis (15, 23). Therefore, myosteatosis may be useful for quantitatively assessing a common clinical phenotype for the overall status of the body, such as metabolic status, gene status, obesity, inflammation and so on, which is thought to underlie variability in responses to immune-HAIC therapy and prognosis in HCC. On the one hand, this pathological linkage might have played a role in the induced effectiveness of immune-HAIC treatment in HCC. Several studies have suggested that patients’ metabolic state is a critical modulator of the effectiveness of immune checkpoint inhibitors (24). Turner et al. revealed a strong association between pembrolizumab (a kind of ICI) clearance and prognosis. Patients with high pembrolizumab clearance rates showed significantly lower survival rates compared to that in patients with low clearance rates, suggesting that the major catabolic pathway of pembrolizumab could be influenced by factors that might also be involved in the development of cancerous cachexia, including myosteatosis. Therefore, myosteatosis patients with muscle weakening/wasting in our cohort might have resulted in a high nivolumab clearance status, which could partly be responsible for a worse therapeutic response and prognosis. Hence, myosteatosis with poor muscle quality in this cohort of patients could be due to metabolic disturbances, which are further associated with an increased risk of worse treatment response and survival time. Since many patients may have simultaneous muscle mass loss (sarcopenia), relative measurements of the body composition changes may facilitate or guide the effectiveness of ICI treatment. On the other hand, we believe the potential for improving interpretability of body composition features, such as myotsteatosis for prediction of treatment response may be assessed in several ways. First, correlation matrix evaluation may be performed between myosteatosis and clinical parameters which may be associated with treatment response in HCC, such as serum creatinine level, albumin (25), etc. This approach may help to understand how body composition features may be associated with the routine clinical features that are commonly used in clinical practice. Second, body composition features may reflect the biological behavior of the tumor such as tumor aggressiveness status, which should help to predict response to treatment.

The performance of our prediction models also reflected the role of myosteatosis as a predictor for treatment response and prognosis. Upon the addition of SM-RA, the efficacy of our combined prediction model was significantly improved for predicting therapeutic efficacies in HCC patients. In addition, the modest differentiation efficiency of our model was also attributed to the introduction of several clinical and radiological parameters, including RBC, HGB, Scr, and VFmean, whose potential prognostic roles were investigated in previous studies. For example, RBC (26) and HGB (27) have been shown to be critical risk factors for poor prognosis in HCC patients. Relatively reduced Scr level in the Non-OR group when compared to that in the OR group suggested a significant loss of skeleton muscle mass (sarcopenia), which was clearly visible in our cohort and was reported to be associated with the poor prognosis in HCC. As a potential marker for visceral fat inflammatory response, increased VFmean could be associated with a higher risk of treatment failure and poor prognosis in HCC patients. A recent study has reported that elevated visceral fat inflammation is frequently observed in HCC patients indicating the possibility of an unfavorable prognosis. Therefore, it was not surprising to notice that the model performance was significantly improved after incorporating these known etiological factors associated with unfavorable outcomes.

RAS mutation is the second most important oncogenic driver in liver carcinoma (28, 29). KRAS mutations are pathologically linked to the increased expression of PD-L1 (30), a known predictor of treatment response to ICIs. However, some of the recent studies have suggested that patients with activating mutations in the *KRAS* gene may benefit from PD-1 blockade (31, 32). Interestingly, we revealed a negative correlation between the VFmean and *KRAS* mutations in this study. However, it is still uncertain whether there is a causal relationship between these two factors. Further investigations are warranted to determine the association between the VFmean and *KRAS* mutation.

There are a few limitations to this study. First, it was a single-centered retrospective study that might involve the case selection bias. Second, despite the inclusion of a relatively large number of OR patients, the number of cases with Non-OR was relatively small, which could have affected the performance of the predictive models as well as the generalizability of our study results. Third, our research lacks external validation, which may potentially affect the generalization ability of our results. Large-scale multicenter prospective studies with a standardized imaging protocol and the standard body composition measurement tool should be important to validate our results.

## Conclusions

Measurements of CT-derived myosteatosis could be associated with a higher risk of treatment failure and poor prognosis in HCC patients undergoing immune-HAIC therapy. The myosteatosis nomogram constructed in this study could potentially support an individualized prediction of treatment response and prognosis, thus assisting in making treatment decisions for HCC patients before the initiation of immune-HAIC. This study revealed potential clinical uses of the body composition analysis in the overall assessment of disease outcomes in HCC patients.

## Data Availability Statement

The original contributions presented in the study are included in the article/supplementary material. Further inquiries can be directed to the corresponding author.

## Ethics Statement

The studies involving human participants were reviewed and approved by the Medical Ethics Committee of the Xiangya Hospital. The patients/participants provided their written informed consent to participate in this study. Written informed consent was obtained from the individual(s) for the publication of any potentially identifiable images or data included in this article.

## Author Contributions

All authors listed have made a substantial, direct and intellectual contribution to the work, and approved it for publication.

## Funding

This work was supported by grants from the National Natural Science Foundation of China (No. 81773234) and Scientific Research Project of Hunan Health and Health Commission (No. C2019189).

## Conflict of Interest

Author HL was employed by GE Healthcare.

The remaining authors declare that the research was conducted in the absence of any commercial or financial relationships that could be construed as a potential conflict of interest.

## Publisher’s Note

All claims expressed in this article are solely those of the authors and do not necessarily represent those of their affiliated organizations, or those of the publisher, the editors and the reviewers. Any product that may be evaluated in this article, or claim that may be made by its manufacturer, is not guaranteed or endorsed by the publisher.
